# Nutritional Composition of Plant Protein Beverages on China’s Online Market: A Cross-Sectional Analysis

**DOI:** 10.3390/nu15122701

**Published:** 2023-06-09

**Authors:** Jialin Zhang, Qiang Cai, Wei Ji

**Affiliations:** Yangtze Delta Region Institute of Tsinghua University, Jiaxing 314000, China; zhangjialin@tsinghua-zj.edu.cn (J.Z.);

**Keywords:** plant protein beverage, plant milk, protein, nutritional composition, coconut, soy, oats

## Abstract

Plant protein beverages are gaining popularity due to various reasons such as lactose intolerance, veganism and health claims. This study aimed to conduct a cross-sectional analysis of plant protein beverages sold online in China, with a focus on assessing their nutritional composition. A total of 251 kinds of plant protein beverages were analyzed, including coconut (*n* = 58), soy (*n* = 52), oats (*n* = 49), walnut (*n* = 14), almond (*n* = 11), peanut (*n* = 5), rice (*n* = 4), other beans (*n* = 5), mixed nuts (*n* = 5) and mixed beverages (*n* = 48), according to the nutrition label on the commercial package and retailer websites. The results showed that, except for soy beverages, plant protein beverages generally had low protein content, cereal beverages showed relatively high energy and carbohydrate levels, and all plant protein beverages had low sodium content. Additionally, the fortification rate of vitamins and minerals in the analyzed plant protein beverages was found to be extremely low, at only 13.1%. Given the substantial variation in the nutritional composition of plant protein beverages, consumers should pay more attention to the nutrition facts and ingredient information when choosing these beverages.

## 1. Introduction

The global market for plant protein beverages is expanding rapidly. According to a comprehensive study by Market Research Future (MRFR), the plant protein beverage market is expected to exceed USD 388.42 billion by the end of 2027 [1]. This trend is also reflected in the Chinese market, as evidenced by data from leading e-commerce platforms. According to Tmall’s “2020 Plant Protein Beverage Innovation Trend” report, the market of plant protein beverages experienced an 800% growth rate in 2020, with a 900% increase in the number of buyers. Furthermore, data from JD Supermarket indicated a 77% year-on-year increase in plant protein beverage sales turnover in 2021. However, compared to Western countries, the Chinese plant-based market is still in its early stages of development [2].

The rising popularity of plant protein beverages among consumers can be attributed to various reasons, including milk allergy, lactose intolerance, hypercholesterolemia [3], vegetarianism [4] and environmental concerns [5]. The prevalence of lactose intolerance in Asian countries further emphasizes the importance of lactose-free alternatives [6,7]. Moreover, consumers perceive plant protein beverages as a nutritious option [8,9]. However, it is worth noting that consumers’ knowledge regarding plant protein beverages in China remains limited, despite a high purchasing rate [10]. Consequently, a comprehensive analysis of plant protein beverages is warranted to inform consumers and address their nutritional requirements.

Plant protein beverages offer diverse health-promoting properties and functional substances derived from different plant sources, meeting consumers’ expectations of natural health, multiple health functions and the presence of multivitamins [11]. For example, soybeans contain isoflavones with cancer prevention [12] and anti-aging effects [13]. Oats contain oat anthracamide, an alkaloid not found in other grains, which exhibits antioxidant and anti-inflammatory activities [14]. Lauric acid in coconut is related to brain development promotion [15] and increased levels of high-density lipoprotein cholesterol [16]. Walnuts are considered a food with both medicinal and dietary characteristics in China, known to improve blood lipids, immunity and brain function [17,18]. These positive health effects mainly stem from the presence of rich, unsaturated fats in walnuts, such as linolenic acid and oleic acid. Understanding the unique nutritional profiles and potential health benefits of different plant protein beverages is crucial for consumers seeking specific health outcomes.

Typically, plant protein beverages are classified into five main categories: cereals, legumes, nuts, seeds and pseudocereals [19,20]. In the Chinese market, common plant protein beverages can be broadly categorized into four groups: cereals, legumes, nuts, fruits and vegetables. The nutritional differences among plant protein beverages are significant due to variations in plant sources, production processes, nutritional fortification and other factors [8,21]. However, many consumers are unaware of these differences [22,23]. A Swiss study revealed that, except for soy beverages, dairy milk contains more protein than most non-dairy plant-based beverages, and only a few non-dairy plant-based beverages contain calcium and vitamins [24]. Studies conducted in the United States, Australia and Europe have also shown regional disparities in nutritional fortification, with the United States having the highest nutritional fortification rate [25]. To our knowledge, there is a lack of analysis on Chinese plant-based beverage products.

The objective of this study was to conduct a comprehensive cross-sectional analysis of plant protein beverages available for online purchase in China. We collected information on the nutritional composition, fortification and additives of plant protein beverages. We aimed to explore the nutritional differences among plant protein beverages in China and to understand whether they are additionally fortified. Information on sugar, cholesterol, trans fat and dietary fiber, which are of interest to consumers, was also recorded to analyze the health status of each plant protein beverage. By providing valuable insights into the Chinese market, this research aims to contribute to the understanding of plant protein beverages and their role in meeting consumers’ nutritional needs.

## 2. Materials and Methods

### 2.1. Data Collection

Between July and October 2022, data collection was conducted on China’s prominent online shopping platforms, namely Taobao and JD.com. A keyword-based search approach was employed to screen and collect products that met the criteria of plant protein beverages. A product was considered a plant protein beverage if it met either of the following conditions: (1) the packaging was labeled as a “plant protein beverage”, or (2) the product’s execution standard identified it as a “plant protein beverage”. Nutrient composition and relevant information were extracted from the nutrition label on the commercial packaging or provided by retailers on their websites. The mandatory nutrient information in China includes energy, protein, fat, carbohydrate and sodium. Hence, the collected data included brand, origin, ingredients, implementation standards, quality guarantee period, net content, energy, protein, fat, carbohydrate and sodium. All products meeting the inclusion criteria, except those originating from outside China or lacking the aforementioned information, were included in the analysis. Additionally, voluntarily marked nutrients on the packaging, such as saturated fats, trans fats, cholesterol, dietary fiber, sugar, lactose, minerals (calcium, iron, potassium, magnesium, phosphorus, selenium, zinc) and vitamins (niacin, vitamin A, vitamin B2, vitamin B6, vitamin D, vitamin E), were also collected. Initially, a total of 288 products were collected, and 31 products containing powdered milk were excluded from the preliminary analysis. Subsequently, the products were grouped by their plant protein sources, such as coconut, soy, oats, etc. Groups with fewer than four brands were excluded from the analysis. The final dataset for analysis comprised 251 products from 138 brands in China.

### 2.2. Evaluation Criterion

The evaluation of plant protein beverages was conducted based on the Nutrient Reference Values (NRV) in China, which are derived from the recommended nutrient intake (RNI) and the appropriate intake (AI) of Chinese residents. For every 100 mL of beverage, according to Chinese pre-packaged food nutrition standards [26], the following criteria were used: protein ≥ 3.0 g was considered a significant protein source; fat ≤ 1.5 g met the low-fat standard; sodium ≤ 120 mg met the low-sodium standard; sodium ≤ 40 mg met the extremely low-sodium standard; fat ≤ 0.75 g met the low saturated fat standard; dietary fiber ≥ 1.5 g indicated a beverage as a source of dietary fiber, and dietary fiber ≥ 3 g indicated a beverage rich in dietary fiber. Regarding vitamins and minerals, ≥7.5% NRV indicated that the beverage was a source of vitamins and minerals, and ≥15% NRV indicated that it was rich in vitamins and minerals. The specific values corresponding to 7.5% and 15% NRV for vitamins and minerals are shown in Table 5.

Plant protein beverages are not a major source of daily energy for the body, and for energy assessment we considered a bottle of plant protein beverage with no more than 6% NRV. Most of the plant protein beverages in our survey were 250 mL per bottle. Therefore, for every 100 mL of the beverage, the energy standard was set at 201.6 kJ. As carbohydrates supply 60% of energy [26], the carbohydrate standard was established at 7.2 g. By combining the protein sources, low-fat and extremely low-sodium standards, the nutritional value of the beverages was evaluated using the following criteria for every 100 mL beverage: energy content ≤ 201.6 kJ; protein ≥ 3 g; fat ≤ 1.5 g; carbohydrate ≤ 7.2 g; and sodium ≤ 40 mg.

### 2.3. Statistical Analysis

SPSS, version 25 (IBM Corp, Armonk, NY, USA, released 2017) was used for data analyses. For the data analysis of energy, protein, fat, carbohydrate and sodium in vegetable protein drinks, Kolmogorov–Smirnov was used to test whether the data obeyed normal distribution. Descriptive statistics, including the median and interquartile ranges, were used to summarize the data after categorizing the products based on their respective plant-based types. As the data did not follow a normal distribution, the Kruskal–Wallis test was used to examine the differences in nutrient composition among the various plant-based products. Subsequently, the Bonferroni method was applied for multiple comparison adjustments, with a significance level of *p* < 0.05 being considered statistically significant.

The number and percentage of plant protein beverages that met specific nutritional requirements and were fortified with nutrients are detailed. Specific nutritional requirements included energy ≤ 201.6 kJ; protein ≥ 3 g, fat ≤ 1.5 g, carbohydrate ≤ 7.2 g, sodium ≤ 40 mg, trans-fat-free, cholesterol-free, sugar-free, lactose-free, dietary fiber ≥ 1.5 g, dietary fiber ≥ 3.0 g. Nutritional fortification included Ca, Fe, K, Mg, P, Se, Zn, Niacin, VA, VB2, VB6, VD and VE.

## 3. Results

A total of 251 plant protein beverages were analyzed and classified into different categories: coconut (*n* = 58), soy (*n* = 52), oats (*n* = 49), walnut (*n* = 14), almond (*n* = 11), peanut (*n* = 5), rice (*n* = 4), other beans (*n* = 5), mixed nuts (peanut + walnut/almonds, *n* = 5) and mixed beverages (*n* = 48). The category of “other beans” included mung beans (*n* = 3), red beans (*n* = 1) and chickpeas (*n* = 1). Mixed beverages contained a combination of at least two kinds of plant protein from beans, grains, nuts and coconuts. The net content of a single beverage was generally 250 mL (*n* = 92, 36.65%).

The nutritional components in different types of plant protein beverages expressed as median and range per 100 mL are presented in Table 1. The nutritional composition varied significantly among the different plant protein beverages (*p* < 0.05). Oat beverages had significantly higher median values of energy (246.0 kJ/100 mL) and carbohydrate (7.0 g/100 mL) compared to other beverages. Rice beverages also had relatively high energy and carbohydrate levels, with no significant differences observed among other beverages. There were significant differences in protein content, with soy beverages having the highest value (3.0 g/100 mL), while the remaining beverages had median values ranging from 0.6 to 1.2 g/100 mL. Coconut (2.6 g/100 mL), oats (2.6 g/100 mL) and walnuts (2.5 g/100 mL) were high in fat. The sodium level of nut beverages was higher than that of others, with mixed nuts beverages having the highest sodium content of 65.0 mg/100 mL.

Table 2 displays the number (%) of plant protein beverages per 100 mL based on the suggested nutrient guidelines. Nearly 80% of oat beverages exceeded the recommended energy limit of 201.6 kJ/100 mL. Except for oat and mixed beverages, at least 50% of the other beverages had an energy content lower than or equal to 201.6 kJ/100 mL. Only 13.9% of the beverages contained at least 3 g protein/100 mL, and the only plant-based types which met this guideline were soy (53.9%) and mixed beverages (14.6%). Twenty-nine point five percent of beverages had no more than 1.5 g fat/100 mL. Coconut (1.7%) and walnut (0.0%) drinks had higher fat contents than 1.5 g/100 mL, while rice and other bean beverages had lower fat contents than 1.5 g/100 mL. Seventy-nine point three percent of beverages contained no more than 7.2 g carbohydrates/100 mL. Sixty-eight point five percent of the beverages contained no more than 40 mg sodium/100 mL. Peanuts, walnuts and mixed nuts had the lowest proportion of beverages that met extremely low sodium standards. We used five specific nutritional requirements in Table 2 as screening criteria simultaneously, and three soybean beverages met all standards.

Out of the analyzed products, 65 (25.9%) products were labeled as trans-fat-free, with oats (38.8%) and mixed beverages (41.7%) being the most common. Seventy-nine (31.5%) products were labeled as cholesterol-free, with walnuts (57.1%) being the most prevalent. Thirty-nine (15.5%) products were marked as sugar-free. Plant protein beverages generally do not contain lactose, but only 11 (4.4%) products were marked as lactose-free, and most plant-based were not tagged as lactose-free. Rice was the only beverage without any “free” labelling (Table 3).

Among the 251 beverages, dietary fiber content was indicated in 61(24.3%) of them, with oats having the highest identification rate of 67.4%. Among the beverages labeled with dietary fiber content, 28 (49.5%) contained at least 1.5 g of dietary fiber per 100 mL, and 13 (21.3%) had at least 3.0 g of dietary fiber per 100 mL (Table 4). The source of dietary fiber in plant protein beverages is not only the plant itself; some beverages have added polydextrose, oligofructose and resistant dextrin to enhance the dietary fiber content of the beverage.

The labeling of vitamins and minerals is not mandatory in China. In this study, a majority of the beverages did not have labels indicating the content of vitamins and minerals. We have conducted statistical analysis on all vitamins and minerals indicated in the beverage nutrition table, where only 33 (13.1%) plant protein beverages had labels indicating the presence of vitamins or minerals. Among these, 25 (10.0%) beverages were labeled with mineral content, and 12 (4.8%) beverages were labeled with vitamin content. Regarding the amount of labeled minerals, calcium fortification was relatively common, with 17 beverages containing at least 60.0 mg/100 mL of calcium and 14 beverages containing at least 120.0 mg/100 mL. As for vitamins, vitamin E fortification was the most prevalent, with five out of seven beverages containing at least 1.1 mg of alpha-TE vitamin E per 100 mL (Table 5).

To prevent the occurrence of beverages that are fortified but not labeled in terms of content, the actual fortification rates of calcium, vitamin D, vitamin B2, vitamin B12 and vitamin E, which are often added to plant protein beverages, were detailed through an analysis of additives in the ingredient list. Twenty-two (8.8%) of the beverages were calcium-fortified, mostly with calcium carbonate and tricalcium phosphate. As food additives, calcium carbonate and tricalcium phosphate have roles as acidity regulators and stabilizers in addition to calcium fortification. Two beverages were fortified with vitamin D, three beverages were fortified with vitamin B2, no beverages were fortified with vitamin B12, and nine beverages were fortified with vitamin E. The actual nutritional fortification rates of the plant protein beverages we included in our analysis were generally consistent with the labeling rates.

## 4. Discussion

### 4.1. Protein, Energy, Carbohydrate, Fat and Sodium

The comparison of the nutritional characteristics of plant protein beverages shows that there are significant differences in the nutritional value of different plant protein beverages, highlighting the importance of considering these differences when choosing such products. Our findings align with previous studies, further confirming the variations in nutritional value among different plant protein beverages [27].

One notable limitation of plant protein beverages is their generally low protein content, as observed in most commercially available products [28]. Our analysis also showed significant differences in protein levels among plant protein beverages, with soy beverages having the highest protein content of 3.0 g/100 mL, surpassing other plant protein beverages. Except for soy and mixed beverages, the protein content in other beverages ranged from 0.0 to 1.8 g/100 mL. Consistent with previous studies, only soy beverages achieved protein levels comparable to milk [27,29]. These results reinforce the importance of considering protein levels when choosing plant protein beverages, particularly for individuals with higher protein requirements.

The variability in protein content among plant protein beverages can be attributed to the use of different plant sources and the presence of plant-based additives. Soy, with its high protein content of 36.5 g/100 g [29], outperformed other plant sources such as oat, almond and coconut. Additionally, the presence of plant-based additives, which can range from 2% to 12% [30], also contributed to the observed variations in protein levels.

The energy and carbohydrate content of drinks is of concern to the weight loss and fitness crowd. Energy and carbohydrate levels were also examined in this study. Grain beverages (oats, rice) typically contain additional carbohydrates in the form of starch [30], resulting in higher energy and carbohydrate levels compared to other beverages. Plant protein beverages generally had lower energy levels compared to whole milk, with an average energy of 262 kJ/100 mL [31], primarily due to their lower sugar content [29].

In terms of nutritional labeling, it is important to note that the labeling of sugar, lactose and dietary fiber content is not mandatory in China. However, as consumers become more health-conscious, brands often use “free” labels to attract consumers. Our analysis revealed that 15.5% of plant protein beverages were marked as “sugar-free”. Although most plant protein beverages lack information on sugar content, we further analyzed and explained the components of plant protein beverages. Ingredient analysis indicated that 74.5% of plant protein beverages had added sugar or sweeteners, including non-nutritional sweeteners. Of interest is that about 70% of the 49 oat beverages had no added sugar or sweeteners. It is crucial to be aware that while sugar is commonly used to enhance the sensory quality of plant protein beverages, it may have negative impacts on nutritional quality and blood glucose index [32,33].

One advantage of plant protein beverages over milk is the absence of lactose. However, only 4.4% of plant protein beverages are labeled as “lactose-free” in our study, indicating a need for improved labeling practices to facilitate informed choices for lactose-intolerant individuals. Another advantage is the presence of dietary fiber, which provides various health benefits [32]. Our study revealed that approximately half of the beverages labeled with dietary fiber content contained 1.5 g or more dietary fiber per 100 mL, with oat beverages having the highest labeling rate. This finding highlights the potential of plant protein beverages, particularly those rich in dietary fiber, in promoting intestinal regulation and contributing to the prevention of diseases such as diabetes, cardiovascular disease, gastrointestinal disease and colon cancer [34].

Regarding fat content, all plant protein beverages included in this study had a lower fat content compared to milk, with the average fat content of milk being 3.3 g/100 mL [31]. While 29.5% of the beverages had low-fat content (less than 1.5 g/100 mL), oats, coconut and walnut beverages had relatively higher fat content. Plant protein beverages are rich in unsaturated fatty acids and are cholesterol-free [35]. Nevertheless, a cholesterol-free mark in China requires a cholesterol level test before it can be labeled. A significant percentage of the beverages (31.5%) were labeled as cholesterol-free, and 25.9% were labeled as trans-fatty-acid-free. In comparison to milk, most plant protein beverages contain little or no saturated fat, which is known to increase serum cholesterol levels and the risk of cardiovascular diseases [36,37]. The World Health Organization recommends limiting the intake of saturated fat in a healthy diet. Importantly, all plant protein beverages in this study meet the low sodium standard, alleviating concerns about excessive sodium intake from plant protein beverages, despite the relatively high sodium content in nut-based plant protein beverages.

### 4.2. Nutrition Fortification and Additives

Compared with dairy products, most plant protein beverages have lower vitamin and mineral content [19,38]. To address this, many fortified plant protein beverages include added calcium, vitamin B2, vitamin B12, vitamin D and occasionally vitamin E [30]. A study investigating 148 types of plant milk from the United States, Australia and Europe found that 78% were fortified with calcium, 53% with vitamin D and 41% with vitamin B12. Another analysis by Alex Glover et al. [39] on 57 plant protein beverages in UK supermarkets demonstrated that all beverages were calcium-fortified. However, most products in this study did not indicate the mineral and vitamin content on the outer packaging, with only 13.1% being labeled. We further analyzed the ingredient list of the 251 plant protein beverages and found that the actual nutritional fortification was consistent with the labeling.

Regarding calcium content, most plant protein beverages either lack calcium or have very low levels. The typical calcium fortification level is 120 mg/100 g, which is equivalent to the calcium level in milk [31]. In our study, 20 (8.0%) beverages were fortified with calcium, and 70% of them reached the calcium level found in milk. However, the bioavailability of fortified plant protein beverages may be compromised due to the presence of “anti-nutrients” such as phytic acid, oxalate, lecithin and saponin, which can reduce mineral absorption and digestion in the human body [40,41]. Therefore, despite fortification, plant protein beverages still lack several critical nutrients provided by milk [30].

Plant protein beverages usually include additives such as oil, stabilizers, emulsifiers, flavoring agents, sweeteners and salt [28] to ensure taste and stability. In response to consumer concerns about sugar and health, some products are beginning to use non-nutritive sweeteners such as sucralose and erythritol. However, the health evaluation of sugar substitutes remains controversial [42,43], and the use of sugar substitutes does not necessarily make the products healthier. Emulsifiers are also prevalent additives and include monoglyceride and diglycerol fatty acid esters, sucrose fatty acid esters and sodium caseinate. It is worth noting that sodium caseinate, which comes from milk, was added as an emulsifier in 28.7% of the products, potentially posing health risks to consumers with milk allergy or intolerance symptoms.

With the increasing awareness of consumer health, products are also pursuing a “cleaner” ingredient list [44]. In our study, we identified 17 types of beverages that did not contain any additives except water and plant-based ingredients. These beverages were basically soy drinks, but also included oat and rice drinks. It is worth mentioning that some of these beverages had a shelf-life of up to 12 months, demonstrating their stability and potential for long-term consumption.

### 4.3. Functional Active Substances and Potential Health Benefits

Plant protein beverages contain additives, phenolic compounds, unsaturated fatty acids, antioxidant activity and phytoactive compounds, which contribute to their positive effects [45]. Different plant-based substances exhibit unique functional activities and health benefits, which attract certain consumers [11]. Soy saponins can inhibit the DNA synthesis and cell metastasis of tumor cells [46] and have a variety of physiological activities such as anti-oxidation and anti-hyperlipidemia properties and the inhibition of liver dysfunction. Almonds are rich in vitamin E [20] and selenium and contain amygdalin, a compound unique to apricot plants. Amygdalin has an anti-tumor effect on solid tumors such as lung cancer by influencing the cell cycle and inducing cell apoptosis [47]. Peanuts contain γ- Aminobutyric acid, which has the physiological function of promoting sleep [48] and has been used in beverages and other foods.

However, some health benefits of plant ingredients are also controversial. Soybean isoflavones have been proven to have anticancer [12], heart disease prevention [49], antioxidant and anti-aging [50] effects. However, the relationship between soy isoflavones and breast enlargement and breast cancer risk is still controversial [51,52]. Coconut has a unique nutritional composition and characteristics [20], and it is rich in vitamins and minerals. Coconut has a very high saturated fatty acid content, and Neelakantan et al. [53] argue that coconut oil should not be considered a healthy oil. However, some studies confirm the health benefits of coconut oil related to cardiovascular risks, such as glycemic control and obesity management [54].

## 5. Strengths and Limitations of the Study

This study has several strengths and limitations that should be acknowledged. While we aimed to include a wide range of products available on the Chinese market, it is important to note that some regional-specific products and those not available for online purchase were not included in our analysis. However, online shopping has become the dominant method for Chinese consumers [55], and the plant protein beverages we collected are readily accessible through online platforms. Our sample covered well-known and emerging brands, as well as popular categories of plant milk on the current Chinese market. Before this study, there was a lack of research examining the nutritional status of Chinese plant protein beverages. Therefore, our findings provide valuable insights that can assist consumers in making informed choices and can contribute to the future development of plant protein beverages in China.

Additionally, there are several limitations to consider. Firstly, this study relied solely on data obtained from product packaging and their websites, specifically the mandatory labeled nutritional components. Therefore, there is a possibility of missing data regarding other nutritional components. Furthermore, the absence of information about the amount of plant-based ingredients added to the product packaging hindered our ability to estimate the content of certain nutrients accurately. To address this limitation, we collected and analyzed data from the formula table of the products, which helped to determine the presence of added sugar, vitamins and minerals in the 251 plant protein beverages. In future research, alternative data collection methods, such as the laboratory analysis of product samples, could be employed. This approach would allow for a more comprehensive and detailed assessment of the nutritional composition of plant protein beverages.

## 6. Conclusions

Through the analysis of 251 kinds of plant protein beverages available on the Chinese market, several key findings emerged. Firstly, there was a significant difference in protein levels among beverages, with soybean beverages having significantly higher protein levels compared to other beverages. Cereal beverages (oats, rice) had higher levels of energy and carbohydrate, while coconut, oatmeal and nut beverages were high in fat. The sodium content of nut beverages was relatively high, although all beverages met the low sodium standard for Chinese beverages. Notably, the overall nutritional fortification rate of the analyzed products was extremely low. However, 70% of the calcium-fortified products reached the calcium level found in milk. Most beverages contained added sweeteners and other additives to adjust the taste, whereas 6.8% of the products contained only water and plant-based ingredients without any additional additives.

Although plant protein beverages offer unique nutritional advantages, it is still difficult to completely replace milk, especially for children [56,57]. When choosing plant protein beverages, consumers should pay close attention to the nutritional composition table, ingredient list, and allergy information provided on the product packaging. Individuals with lactose intolerance and milk protein allergies should be aware that some products labeled as “plant protein beverage” or containing “plant protein” in their keywords on shopping websites may still contain milk powder. Therefore, it is essential to carefully review the allergy information indicated on the product packaging. From a protein standpoint, soy beverages are a favorable choice. Furthermore, for taste and nutritional balance, mixed plant protein beverages appear to be a good option. However, it remains crucial to obtain sufficient nutrients from other dietary sources.

## Figures and Tables

**Table 1:** Median (Q1–Q3) values of nutrients in plant protein beverages (per 100 mL) on China’s online market.

Type of Plant Basis	*n*	Energy (kJ)	Protein (g)	Fat (g)	Carbohydrates (g)	Sodium (mg)
Almond	11	160.0 (114.0–207.0) ^b^	1.0 (0.7–1.2) ^b, c, d^	2.1 (1.6–2.2)	4.9 (1.6–6.8) ^b^	40.0 (40.0–51.0) ^a^
Coconut	58	201.0 (169.0–223.2) ^b^	0.6 (0.6–0.7) ^d^	2.6 (2.0–3.0) ^a^	6.4 (4.2–7.0) ^b^	36.0 (23.0–49.8) ^a, c^
Oats	49	246.0 (212.0–270.0) ^a^	1.1 (1.0–1.3) ^b, c^	2.6 (1.5–3.1) ^a^	7.0 (6.5–8.5) ^a^	37.0 (30.0–45.0) ^a, c^
Peanut	5	137.0 (115.0–158.0) ^b^	1.0 (0.8–1.0)	1.6 (1.5–1.8)	3.5 (2.9–4.4) ^b^	52.0 (28.0–52.0)
Rice	4	201.5 (181.5–211.8)	0.6 (0.4–0.6) ^c, d^	1.0 (0.7–1.0)	9.6 (7.9–10.8)	11.5 (0.0–32.2)
Soy	52	195.0 (173.2–208.8) ^b^	3.0 (2.0–3.6) ^a^	1.8 (1.3–2.2) ^b^	4.2 (1.8–6.5) ^b^	20.0 (10.0–35.0) ^b, c^
Walnut	14	192.9 (161.8–217.5)	0.6 (0.6–0.8) ^c, d^	2.5 (2.4–3.1) ^a^	3.9 (3.2–5.7) ^b^	51.0 (26.2–57.2) ^a, c^
Other beans	5	150.0 (128.0–160.0) ^b^	0.9 (0.9–1.0)	0.0 (0.0–1.4) ^b^	6.5 (5.1–8.5)	15.0 (15.0–16.0) ^c^
Mixed nuts	5	143.0 (140.0–151.0) ^b^	0.7 (0.7–0.8) ^b, c, d^	1.4 (1.4–2.0)	4.2 (3.8–4.5) ^b^	65.0 (45.0–66.0) ^a^
Mixed beverages	48	208.0 (156.0–233.7) ^b^	1.2 (1.0–2.3) ^b^	1.8 (1.3–2.8)	5.6 (4.5–6.8) ^b^	29.0 (17.5–40.0)
	*p*	<0.001	<0.001	<0.001	<0.001	<0.001

Various letters exhibited (a, b, c, d) in the same column indicate significant differences in plant-based types. *p* < 0.05 is considered statistically significant.

**Table 2:** Number (%) of plant protein beverages meeting the suggested nutrient guideline per 100 mL.

Type of Plant Basis	*n*	Energy ≤201.6 kJ	Protein ≥3.0 g	Fat ≤1.5 g	Carbohydrates ≤7.2 g	Sodium ≤40.0 mg
Almond	11	7 (63.6%)	0 (0.0%)	3 (27.3%)	10 (90.9%)	7 (63.6%)
Coconut	58	29 (50.0%)	0 (0.0%)	1 (1.7%)	50 (86.2%)	38 (65.5%)
Oats	49	10 (20.4%)	0 (0.0%)	15 (30.6%)	27 (55.1%)	28 (57.1%)
Peanut	5	5 (100.0%)	0 (0.0%)	2 (40.0%)	5 (100.0%)	2 (40.0%)
Rice	4	2 (50.0%)	0 (0.0%)	4 (100.0%)	1 (25.0%)	3 (75.0%)
Soy	52	33 (63.7%)	28 (53.9%)	22 (42.3%)	47 (90.4%)	45 (86.5%)
Walnut	14	7 (50.0%)	0 (0.0%)	0 (0.0%)	12 (85.7%)	6 (42.9%)
Other beans	5	5 (100.0%)	0 (0.0%)	5 (100.0%)	3 (60.0%)	5 (100.0%)
Mixed nuts	5	5 (100.0%)	0 (0.0%)	3 (60.0%)	5 (100.0%)	1 (20.0%)
Mixed beverages	48	21 (43.8%)	7 (14.6%)	19 (39.6%)	39 (81.3%)	37 (77.1%)
	251	124 (49.4%)	35 (13.9%)	74 (29.5%)	199 (79.3%)	172 (68.5%)

**Table 3:** Number (%) of plant protein beverages reaching the “free” standard per 100 mL.

Type of Plant Basis	*n*	Trans-Fat-Free	Cholesterol-Free	Sugar-Free	Lactose-Free
Almond	11	4 (36.4%)	4 (36.4%)	6 (54.5%)	0 (0.0%)
Coconut	58	2 (3.4%)	1 (1.7%)	15 (25.9%)	0 (0.0%)
Oats	49	19 (38.8%)	22 (44.9%)	4 (8.2%)	5 (10.2%)
Peanut	5	0 (0.0%)	1 (20.0%)	1 (20.0%)	0 (0.0%)
Rice	4	0 (0.0%)	0 (0.0%)	0 (0.0%)	0 (0.0%)
Soy	52	15 (28.8%)	21 (40.4%)	3 (5.8%)	1 (1.9%)
Walnut	14	4 (28.6%)	8 (57.1%)	6 (42.9%)	1 (7.1%)
Other beans	5	1 (20.0%)	1 (20.0%)	0 (0.0%)	0 (0.0%)
Mixed nuts	5	0 (0.0%)	2 (40.0%)	0 (0.0%)	0 (0.0%)
Mixed beverages	48	20 (41.7%)	19 (39.6%)	4 (8.3%)	4 (8.3%)
	251	65 (25.9%)	79 (31.5%)	39 (15.5%)	11 (4.4%)

In China, product indicators need to be tested before the corresponding “free” mark can be applied. Trans-fat-free: trans fatty acids ≤ 0.3 g/100 mL; cholesterol-free: cholesterol ≤ 5 mg/100 mL; sugar-free: sugar ≤ 0.5 g/100 mL; and lactose-free: lactose ≤ 0.5 g/100 mL [26].

**Table 4:** Labeling rate of the dietary fiber, and the number (%) of beverages that met the corresponding level (per 100 mL) among beverages labeled with dietary fiber.

Type of Plant Basis	Labeling Rate of Dietary Fiber (%) *n* = 61 (24.3%)	Dietary Fiber ≥1.5 g *n* = 28 (45.9%)	Dietary Fiber ≥3.0 g *n* = 13 (21.3%)
Almond	0 (0.0%)	0 (0.0%)	0 (0.0%)
Coconut	1 (1.7%)	0 (0.0%)	0(0.0%)
Oats	33 (67.4%)	14 (42.4%)	8 (24.2%)
Peanut	0 (0.0%)	0(0.0%)	0 (0.0%)
Rice	0 (0.0%)	0 (0.0%)	0 (0.0%)
Soy	10 (19.2%)	7 (70.0%)	1 (10.0%)
Walnut	1 (7.1%)	1 (100.0%)	1 (100.0%)
Other beans	1 (20.0%)	0 (0.0%)	0 (0.0%)
Mixed nuts	0 (0.0%)	0 (0.0%)	0 (0.0%)
Mixed beverages	15 (31.3%)	6 (40.0%)	3 (20.0%)

The amount (%) of dietary fiber ≥ 1.5 g and dietary fiber ≥ 3.0 g was the percentage in the labeled dietary fiber beverage.

**Table 5:** Number of beverages marked with mineral and vitamin content.

Mineral *n* = 25 (10.0%)			Vitamin *n* = 12 (4.8%)		
Mineral	Level/100 mL	*n*	Vitamin	Level/100 mL	*n*
P (*n* = 2)	≥52.5 mg	1	VE (*n* = 7)	≥1.1 mg α-TE ^a^	5
	≥105.0 mg	0		≥2.1 mg α-TE	1
Mg (*n* = 2)	≥22.5 mg	1	VB2 (*n* = 3)	≥0.1 mg	3
	≥52.5 mg	1		≥0.2 mg	1
K (*n* = 2)	≥150.0 mg	0	NIACIN (*n* = 4)	≥1.1 mg	2
	≥300.0 mg	0		≥2.1 mg	0
Ca (*n* = 20)	≥60.0 mg	17	VB6 (*n* = 2)	≥0.1 mg	2
	≥120.0 mg	14		≥0.2 mg	0
Zn (*n* = 3)	≥1.1 mg	0	VA (*n* = 2)	≥60.0 μg RE ^b^	1
	≥2.3 mg	0		≥120.0 μg RE	1
Se (*n* = 1)	≥3.75 μg	1	VD (*n* = 2)	≥0.4 μg	2
	≥7.5 μg	1		≥0.8 μg	2
Fe (*n* = 1)	≥1.1 mg	0			
	≥2.3 mg	0			

^a^ α-TE: α-tocopherol equivalents; ^b^ RE: Retinol Equivalent.

## Data Availability

Not applicable.
